# Human Cancer Cells Retain Modest Levels of Enzymatically Active Matriptase Only in Extracellular Milieu following Induction of Zymogen Activation

**DOI:** 10.1371/journal.pone.0092244

**Published:** 2014-03-24

**Authors:** Li-Ling Chu, Yuan Xu, Jie-Ru Yang, Yi-An Hu, Hsiang-Hua Chang, Hong-Yu Lai, Chun-Che Tseng, Hue-Yu Wang, Michael D. Johnson, Jehng-Kang Wang, Chen-Yong Lin

**Affiliations:** 1 Department of Pharmacy, Chi-Mei Medical Center, Tainan, Taiwan; 2 Lombardi Comprehensive Cancer Center, Department of Oncology, Georgetown University, Washington, D.C., United States of America; 3 Department of Medicine, National Defense Medical Center, Taipei, Taiwan, ROC; 4 Department of Biology, Carleton College, Northfield, Minnesota, United States of America; 5 Department of Biochemistry, National Defense Medical Center, Taipei, Taiwan, ROC; Stony Brook University, United States of America

## Abstract

The type 2 transmembrane serine protease matriptase is broadly expressed in human carcinomas and hematological cancers. The proteolytic activity of matriptase is a potential target of drugs and imaging probes. We assessed the fate of active matriptase following the induction of matriptase zymogen activation. Exposing eight human carcinoma cells to pH 6.0 buffer induced robust matriptase zymogen activation followed by rapid inhibition of the nascent active matriptase by hepatocyte growth factor activator inhibitor (HAI)-1. Consequently, no enzymatically active matriptase was detected in these cells. Some active matriptase is, however, rapidly shed to the extracellular milieu by these carcinoma cells. The lack of cell-associated active matriptase and the shedding of active matriptase were also observed in two hematological cancer lines. Matriptase shedding is correlated closely with the induction of matriptase activation, suggesting that matriptase activation and shedding are kinetically coupled. The coupling allows a proportion of active matriptase to survive HAI-1 inhibition by rapid shedding from cell surface. Our study suggests that cellular free, active matriptase is scarce and might not be an effective target for *in vivo* imaging and drug development.

## Introduction

Proteases catalyze the breakdown of proteins by the hydrolysis of peptide bonds. Through the regulated cleavage of proteins, proteases are involved in many highly controlled physiological processes, such as DNA replication, cell-cycle progression, cell death, angiogenesis, blood coagulation, inflammation, neurogenesis and immunity. Protease dysregulation has been implicated in a broad range of diseases, including cancer and cardiovascular disorders. Proteases are, therefore, considered to be effective targets for development as drug targets and biomarkers. Proteasome inhibitors, for example, have been used to treat hematological malignancies [1], [2] and serum levels of the protease PSA (prostate specific antigen) have been used as a biomarker for monitoring prostate cancer in various contexts [3]. The invention of activity-based probes (ABP) allows the assessment of protease activity within living cells or in whole organisms [4]. In spite of the success of some drugs and probes, however, targeting proteolytic activity for development of drug and biomarkers has not always been very satisfying. As attractive as they are, proteases-inspired diagnostics and therapies have many inherent complexities and limitations that need to be taken into consideration before developing new drugs or probes targeting proteases and proteases activities. These limitations include the activational status of the proteases, the functional localization of the proteases, and endogenous proteases inhibitors, all of which affect protease activity and can in turn affect the effectiveness of the protease inhibitor and probes.

The type 2 transmembrane serine protease (TTSP) matriptase is a particularly interesting example of the challenges that a protease can present regarding its choice as a target for the development of clinical applications and the strategies that might be required to effectively employ inhibitors of and probes for matriptase activity. Matriptase is broadly expressed by epithelial tissues and indeed is required for the maintenance of epithelial integrity [5]–[7]. Matriptase is commonly dysregulated in carcinomas through elevated expression, increased zymogen activation, and an imbalance in the expression of matriptase relative to hepatocyte growth factor activator inhibitor (HAI)-1, the primary endogenous protease inhibitor of matriptase activity [8]–[10]. In addition to epithelial cells, matriptase is also expressed in monocytes [11]–[13], mast cells [14], chondrocytes [15] and neural progenitor cells [16], and matriptase has been implicated in osteoarthritis [15] and atherosclerosis [13]. The expression of matriptase in mast cells suggests that matriptase has the potential to contribute to allergy-related diseases, such as asthma. Several matriptase catalytic inhibitors have been developed, including small molecule and peptide-based inhibitors. These matriptase inhibitors exhibit great potency against matriptase activity when tested using *in vitro* assays that, in most cases, have made use of recombinant matriptase serine protease domain [17]–[22]. Antibody-based inhibitors specifically targeted against active matriptase (as opposed to the zymogen form) have also been developed [23] and used to detect tumors in mice via binding to active matriptase on the surface of cancer cells [24], [25].

Matriptase is synthesized as a zymogen and undergoes autoactivation to acquire its potent trypsin-like activity. The activation of matriptase is rapidly followed by the inhibition of the nascent active matriptase by the protein HAI-1 and remains attached to the cells through the transmembrane domain of HAI-1. It is unclear how much and for how long nascent free active matriptase persists on the cell surface: parameters that are important for any justification for the development of matriptase activity-based inhibitors and probes for clinical applications. In the current study, we set out to assess the fate of active matriptase following induction of matriptase zymogen activation in human carcinoma and hematological cancer cells. Regardless of the extent of matriptase zymogen activation induced, no free, active matriptase was found to persist on the cancer cells. Interestingly, however, a small proportion of the active matriptase survives HAI-1 inhibition by being rapidly shed into the extracellular milieu. Our study suggests that due to the lack of free active matriptase present on the surface of cancer cells, matriptase catalytic activity is unlikely to present an effective target for clinical applications.

## Materials and Methods

### Chemicals and reagents

Gelatin and 5,5′-Dithio-bis-(2-Nitrobenzoic Acid) (DTNB) were obtained from Sigma-Aldrich (St. Louis, MO); N-tert-butoxycarbonyl (Boc)-Gln-Ala-Arg-7-Amido-4- methylcoumarin (AMC) was purchased from Enzo Life Sciences (Farmingdale, NY); Fetal bovine serum (FBS) was obtained from Omega Scientific (Tarzana, CA).

### Cell cultures

Human breast cancer cells MCF7 and MDA-MB-468 were maintained in a modified Improved Minimum Essential Medium (IMEM), supplemented with 10% FBS. Human prostate cancer cells LNCaP, PC3, and DU145, human ovarian cancer cells OVCAR-3, human multiple myeloma cells RPMI 8226 cells and human Burkitt lymphoma cells Ramos were cultured in RPMI-1640 medium, supplemented with 10% FBS. Human breast cancer cells SK-BR-3, human keratinocyte HaCaT, and human ovarian cancer cells OV-2008 were maintained in Dulbecco's Modified Eagle's medium (DMEM), supplemented with 10% FBS. The cells were incubated at 37°C in a humidified atmosphere with 5% CO_2_. All the cells were obtained from American Type Culture Collection (Manassas, Virginia) except HaCaT (CLS Cell Lines Service GmbH, Eppelheim Germany) and OV2008 (kindly supplied by Dr. Gaetano Marverti, University of Modena and Reggio Emilia, Italy) [26].

### Monoclonal antibodies

The human matriptase monoclonal antibodies M24 and M69 were used for immunoblot analyses to detect total matriptase and activated matriptase, respectively [27]–[29]. The mAb M69 immobilized on Sepharose beads was used for the immunodepletion studies.

### Tryptic activity assay following induction of matriptase zymogen activation

For the induction of matriptase zymogen activation in carcinoma cells, the cells were cultured in 150 mm dishes or 6-well plates. The cells were washed with PBS three times and then incubated for 20 minutes at room temperature with 150 mM phosphate buffer pH 6.0 (7 ml for 150 mm dishes and 1 ml for 6-well plate) or 150 mM phosphate buffer pH 8.0 as a non-activation control. In some cases, PBS was used as the non-activation control. After the incubation, 2 M Trizma base was added to bring the pH to 8.0. The cells were scraped from the dishes or wells to yield a combined suspension containing a mixture of the cells and shed proteins. A proportion of this mixture was subjected to centrifugation at 10,000 rpm using an Eppendorf tabletop centrifuge for 1 min. The supernatant was collected as the shed fraction and the cell pellet was resuspended in 150 mM phosphate buffer pH 8.0 to the same volume as the sample prior to the centrifugation to yield the cell fraction. The procedure of scrapping of the cells from the disk and processing by centrifugation dose not result in the release of matriptase or HAI-1 from the cells. The procedure was designed to generate the cell and conditioned buffer under identical conditions, with respect to pH and ionic strength in the buffer for the proteolytic assay. For the two hematological cancer cells which grow as suspension cultures, the ratio of cell number relative to the volume of the phosphate buffer was 5×10^5^ cells/200 μl buffer. The matriptase activity in 195 μl of the shed and cell fractions was assessed by measuring 7-Amino-4-methylcoumarin (AMC) release from a synthetic substrate Boc-Gln-Ala-Arg-AMC (5 μl of a 5 mM stock solution) in microfluor 96-well black microtiter plates (Thermo Scientific). The cleavage of the synthetic fluorogenic substrate was recorded using a Wallac 1420 Victor 2 microplate reader with an excitation wavelength of 360 nm and detecting emission at 480 nm.

### Immunodepletion

The M69 mAb was covalently coupled to Sepharose 4B at 5 mg/ml gel as described previously [28]. For immunodepletion, the samples (200 μl) were incubated with 15 μl of M69-Sepharose rotating in cold room for 2 hours. The supernatant was separated from the beads by centrifugation and collected as the unbound fraction. The beads were washed with phosphate buffered saline (PBS) containing 1% Triton X100 four times after which the captured proteins were eluted from the beads with 0.1 M glycine buffer pH 2.4, followed by neutralization with 2 M Trizma base.

### Western blotting

Cells were lyzed in PBS containing 1% Triton X-100 and 1 mM DTNB. DTNB was added to the lysis buffer to prevent cleavage of disulfide linkages [30]. The protein concentration was determined by Bradford protein assay and equal amounts of proteins or equal proportions of cell lysates and conditioned buffers were analyzed by Western blot. Protein samples were diluted in 5× sample buffer containing no reducing agent and incubated at room temperature for 5 min. Proteins were resolved by 7.5% SDS-PAGE, transferred to nitrocellulose membranes, and then probed with the various mAbs. The binding of mAbs was detected using HRP conjugated secondary antibodies, and visualized using Western Lightening® Chemiluminescence Reagent Plus (Perkin-Elmer, Boston, MA).

### Gelatin zymography

Gelatin gels were cast by adding gelatin (final concentration 1 mg/ml) to 7.5% SDS polyacrylamide gels. Protein samples were incubated with SDS sample buffer at room temperature for 5 min in the absence of a reducing agent. After electrophoresis, the gelatin gels were washed with 2.5% Triton X-100/PBS three times and then incubated in100 mM Tris buffer pH 8.0 at 37°C overnight. The gels were then stained with Coomassie Brilliant Blue.

## Results

### MCF7 breast cancer cells constitutively activate matriptase but the free active matriptase does not remain associated with the cells

**Figure 1:**
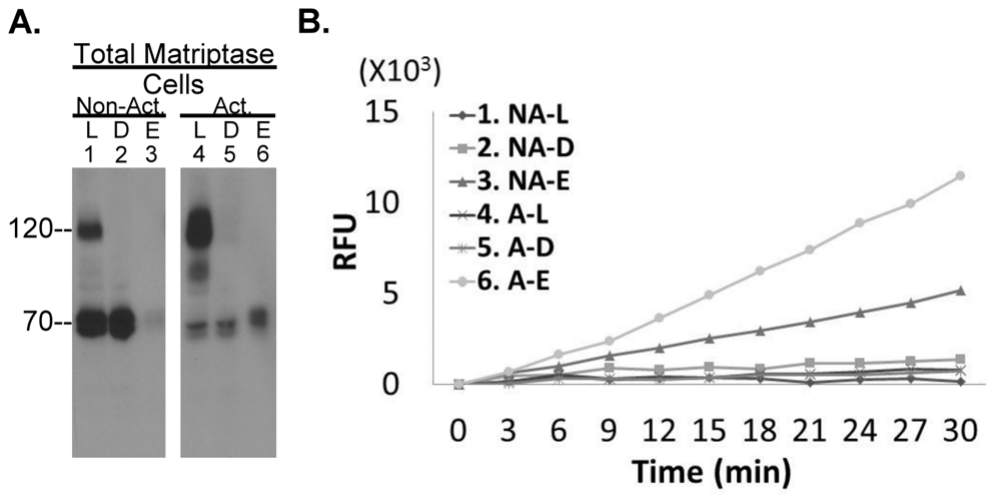
MCF breast cancer cells don't retain enzymatically active matriptase. *A.* MCF7 human breast cancer cells were incubated with a pH 6 buffer to induce matriptase zymogen activation (*A.* right, Act.) or with the pH 6 buffer supplemented with 150 mM NaCl as a non-activation control (*A.* left, Non-act.). Cell lysates were prepared, and samples retained for analysis (lanes 1 and 4). The remaining cell lysates were subjected to immunodepletion with the active matriptase-specific mAb M69 immobilized on Sepharose beads to deplete the activated matriptase, predominantly the 120-kDa complex (lanes 2 and 5, and D). The antibody-bound activated matriptase was recovered by a pH 2.4 buffer elution followed by pH neutralization (Lane 3, E). These samples were then analyzed for matriptase species by SDS-PAGE (without boiling the samples or using reducing agents) and Western blot using the total matriptase mAb M24. *B.* The cells, the immunodepleted lysates, and the eluates were assayed tryptic activity by cleavage of a synthetic fluorogenic substrate with Arg as P1 site. For the tryptic assay, the cells remained intact in the absence of Triton X-100. NA stands for non-activation; L for loading of immunodepletion, D for immunodepleted fraction; E for eluted fraction; A for activation; RFU for relative fluorescent unit.

In order to begin to explore whether matriptase proteolytic activity associated with breast cancer cells might be used as a target for the development of drugs and/or imaging probes, we measured the cleavage of the substrate Boc-Gln-Ala-Arg-AMC by MCF7 breast cancer cells prior to and following the induction of robust matriptase zymogen activation (Fig. 1A). Matriptase zymogen activation apparently occurs constitutively in MCF7 cells since the 120-kDa matriptase-HAI-1 complex was readily detected in addition to the 70-kDa matriptase zymogen in cell lysates prior to the induction of matriptase zymogen activation (Fig. 1A lane 1). After the induction of matriptase zymogen activation by exposure of the cells to pH 6 buffer for 20 min [27], [31], [32], much of the 70k-Da matriptase zymogen was converted to the 120-kDa matriptase-HAI-1 complex (Fig. 1A lane 4). We next measured the amount of tryptic activity associated with MCF7 under constitutive or acid-induced conditions which yields the two different levels of matriptase activation. Given the possibility that HAI-1 might bind to and inhibit the active matriptase after both proteins had been liberated from cell membrane, we measured the tryptic activity associated with the surface of the cells in the absence of the non-ionic detergent Triton X-100. Under these conditions there was barely any cleavage of the fluorescent substrates by the MCF7 cells whether matriptase activation was constitutive or robustly induced by acid exposure (Fig. 1B, curve 1 and 4). These data suggest that these breast cancer cells do not retain free, active matriptase regardless of the levels of matriptase zymogen activation.

The lack of free, active matriptase associated with the cells is due to the rapid inhibition of active matriptase by HAI-1. Previously, we generated a matriptase monoclonal antibody that specifically recognizes and binds to the activated form of matriptase (whether or not it is in a complex with HAI-1), without cross-reacting with matriptase zymogen [33], [34]. We used this mAb, M69, coupled to beads, to remove any activated matriptase that was present in the cell lysates by immunodepletion. As expected, the 120-kDa matriptase-HAI-1 complex was efficiently removed from the MCF7 cell lysates by this process (Fig. 1A, comparing lanes 2 and 5 with lanes 1 and 4, respectively). The 70-kDa matriptase protein band present in both samples was not depleted by the activated matriptase mAb, indicating that the 70-kDa matriptase protein band in both preparations contains little or no active matriptase and is matriptase zymogen. As expected, the immunodepleted samples had no cleavage activity against the fluorescent substrate (Fig. 1B). The activated matriptase species bound to the M69 mAb-Sepharose beads used to immunodeplete the samples were eluted using pH 2.4 glycine buffer. We have previously shown that this acidic buffer causes the dissociation of the matriptase-HAI-1 complex [33]. Neutralization typically results in the re-association of some of the HAI-1 and active matriptase leaving the rest of the dissociated active matriptase in the uncomplexed form after neutralization of the eluate, with the result that the active matriptase was detected by Western blot analysis using a total matriptase mAb predominantly as a 70-kDa band. The intensity of this 70-kDa active matriptase band was proportional to the intensity of the 120-kDa matriptase-HAI-1 complex band detected in both situations (Fig. 1A, lanes 3 and 6). This dissociated active matriptase exhibited tryptic activity as assessed by cleavage of the fluorogenic substrate (Fig. 1B, curve 3 and 6). The substrate cleavage rates observed correlated well with the levels of the active matriptase detected by Western blot analysis. These analyses confirm that the 120-kDa cell-associated matriptase species is an inactivated active matriptase complex and that the eluted 70-kDa species is indeed active matriptase.

### A proportion of active matriptase was shed to the extracellular milieu

**Figure 2:**
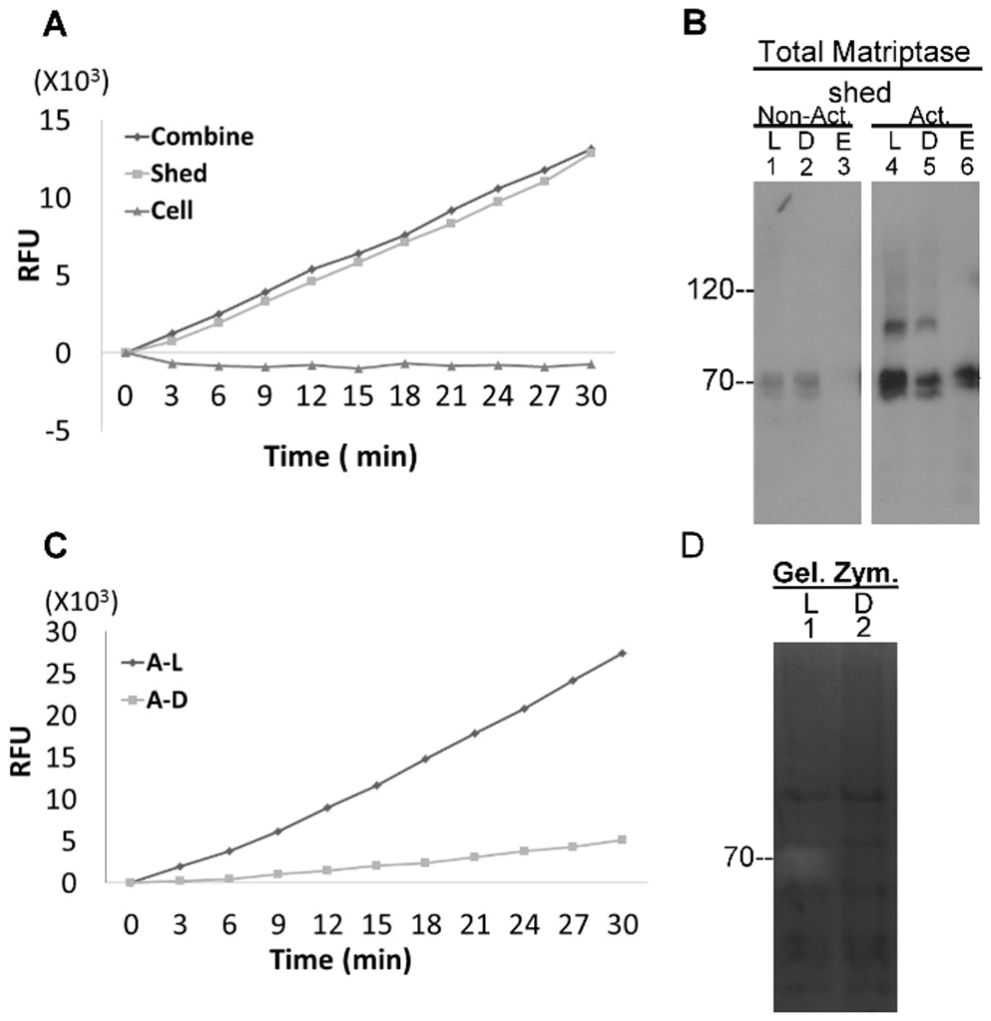
Enzymatically active matriptase is shed to extracellular milieu. *A.* MCF7 cells were induced to activate matriptase by a pH 6.0 buffer followed by neutralization to pH 8.0. The mixture of the cells and the buffer (Combine), the pelleted cells after centrifugation (Cell), and the buffer supernatant (shed fraction) alone (Shed) were analyzed for tryptic activity by cleavage of a synthetic fluorescent substrate with Arg as P1 site. RFU stands for relative fluorescent unit. B. The shed fractions collected from MCF7 cells post induction of matriptase activation (Act.) and the non-activation control (Non-act.) were subjected to immunodepletion with activated matriptase mAb M69 beads. The shed fractions (L, lanes 1 and 4), the depleted fractions (D, lanes 2 and 5) and the eluted fractions (E, lane 3 and 6) were analyzed for matriptase species by Western blot using the mAb M24. *C.* and *D.* The shed fraction collected from pH 6 buffer-exposed MCF7 cells was immunodepleted using M69 beads. The shed fraction (L, lane 1) and the immunodepleted fraction (D, lane 2) were analyzed for the tryptic activity (panel *C*) and gelatinolytic activity by gelatin zymography (panel *D*, Gel. Zym.). RFU stands for relative fluorescent units; A–L for activated-loading; A–D for activated-depleted.

In spite of the rapid HAI-1-mediated inhibition of the active matriptase associated with the cell, free active matriptase was in fact shed into the extracellular milieu prior to HAI-1 inhibition (Fig. 2). When the MCF7 breast cancer cells were transiently exposed to pH 6.0 buffer followed by neutralization to pH 8.0, strong tryptic activity against the fluorescent substrate was detected in the mixture of the cells and conditioned buffers (shed fraction) (Fig. 2A). When the cells and the shed fractions were separated, the tryptic activity was only detected in the shed fractions (supernatant) and not associated with the cell pellets. These data indicate that along with the induction of matriptase zymogen activation, some active proteases with tryptic activity are shed into the extracellular milieu. To address the question of whether this shed proteolytic activity is derived from active matriptase we determined if M69-beads could immunodeplete the activity from the samples (Fig. 2B and C). Immunodepletion of the material shed from the control-treated cells did not result in a significant change in the intensity of the 70kDa matriptase band (Fig. 2B, lanes 1 and 2) and no matriptase was detected in the material eluted from the beads (Fig. 2B, lane 3), consistent with absence or low level of shed free, active matriptase under these conditions. In contrast, after acid-induced matriptase activation, depletion with the M69-beads significantly reduced the levels of total matriptase present in the shed fraction (Fig. 2B, compare lane 5 to lane 4), and a strong 70-kDa matriptase band is present in the eluate from the beads (Fig. 2B, lane 6). This is consistent with the presence of free, active matriptase in the conditioned buffer from the cells in which matriptase activation had been induced by acid exposure. When assayed for tryptic activity using the fluorogenic substrate assay we found that proteolytic activity was dramatically reduced after immunodepletion (Fig 2C, compare the curves A–L and A–D), confirming that the majority of the tryptic activity present in the shed fraction was attributable to free, active matriptase. The presence of active matriptase in the shed fraction was further confirmed by detection of a 70-kDa gelatinolytic activity in the shed fraction (Fig. 2D, lane 1) and the depletion of the 70-kDa gelatinase by the activated matriptase mAb beads (Fig. 2D, lane 2). Taken together, these data suggest that while MCF7 breast cancer cells constitutively activate matriptase under normal conditions, the vast majority of the active matriptase is rapidly inhibited by HAI-1 though a small proportion of the active enzyme may be rapidly shed into the extracellular milieu. The shedding of active matriptase to the extracellular milieu in this way is consistent with the detection of active matriptase in the conditioned media of breast cancer cells in our early study [35].

**Figure 3:**
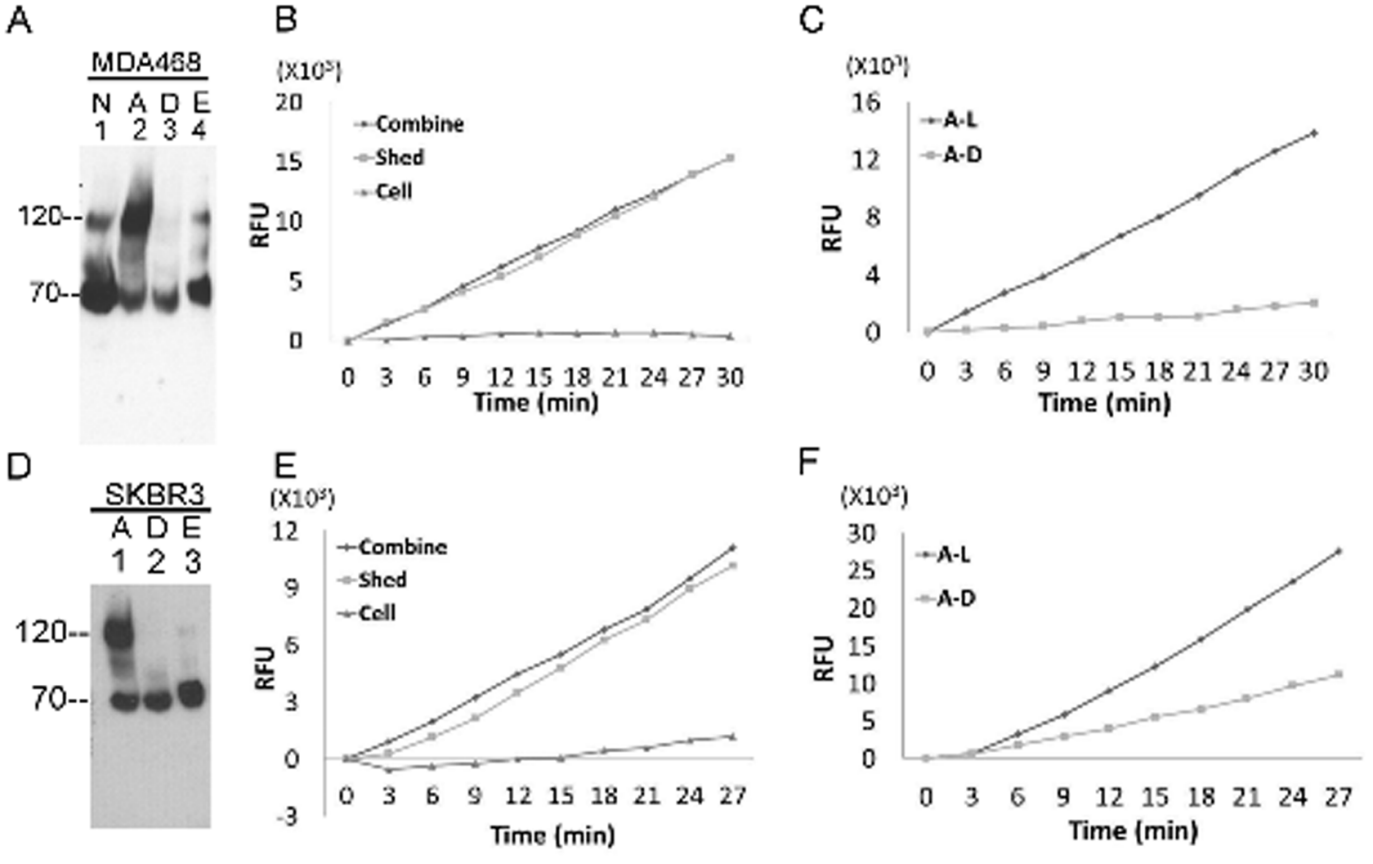
MDA-MB-468 and SK-BR-3 breast cancer cells do not retain free, enzymatically active matriptase on the cell. *A* and *D*: MDA-MB-468 and SK-BR-3 human breast cancer cells were induced to activate matriptase (or not) by pH 6 (or control) buffer-exposure. Cell lysates prepared from the cells were subjected to immunodepletion to remove activated matriptase. Lysates from non-activation control cells (N), acid-activated cells (A), the M69 depleted lysates (D), and the eluted fraction (E) were analyzed for total matriptase species by immunoblot using the matriptase mAb M24. *B* and *E*. MDA-MB-468 (*B*) and SK-BR-3 (*E*) were treated with pH 6.0 buffer to induce matriptase activation. After neutralization, mixture of the cells and the buffer (Combine), the pelleted cells after centrifugation (Cell), and the buffer supernatant (shed fraction) alone (Shed) were analyzed for tryptic activity. *C* and *F*. MDA-MB-468 and SK-BR-3 cell lysates, prepared post induction of matriptase activation were immunodepleted using the mAb M69. The cell lysates (A–L) and the immunodepleted fractions (A–D) were analyzed for tryptic activity.

To determine whether other matriptase-expressing breast cancer cells also regulate matriptase in a similar way to MCF7 cells, we analyzed samples generated using MDA-MB-468 and SK-BR-3 breast cancer cells under normal growth conditions and when subjected to acid-induced matriptase zymogen activation. Matriptase species and proteolytic activity were assessed as before through a combination of fluorescent substrate cleavage assay, immunodepletion and immunoblot analyses (Fig. 3). Similar to MCF7 cells, MDA-MB-468 cells constitutively activate matriptase (Fig. 3A lane 1), and can be induced to robustly activate matriptase followed by rapid HAI-1-mediated inhibition in response to cellular exposure to mildly acidic buffer (Fig. 3A lane 2). The 120-kDa matriptase-HAI-1 complex is specifically immunodepleted using the activated matriptase mAb beads (Fig. 3A lane 3) and the majority of the acid-dissociated free active matriptase was eluted and remained uncomplexed following neutralization (Fig. 3 lane 4). Tryptic activity was detected only in the shed fractions and not in the cell fractions (Fig. 3B), the majority of which was immunodepleted with the activated matriptase mAb M69 (Fig. 3C), confirming the presence of active matriptase in the extracellular milieu. The third breast cancer cells, SK-BR3 resembles MCF7 and MDA-MB-468 with respect to acid-induced matriptase activation (Fig. 3D, lane 1), immunodepletion, and the elution of uncomplexed activated matriptase from the activated matriptase mAb beads (Fig. 3D, lanes 2 and 3). Again, the tryptic activity generated along with the induction of matriptase zymogen activation was shed and not retained with the cells (Fig. 3 E), and the majority of the shed tryptic activity was derived from active matriptase (Fig. 3 F).

### Regulation of matriptase in prostate and ovarian cancer cells

**Figure 4:**
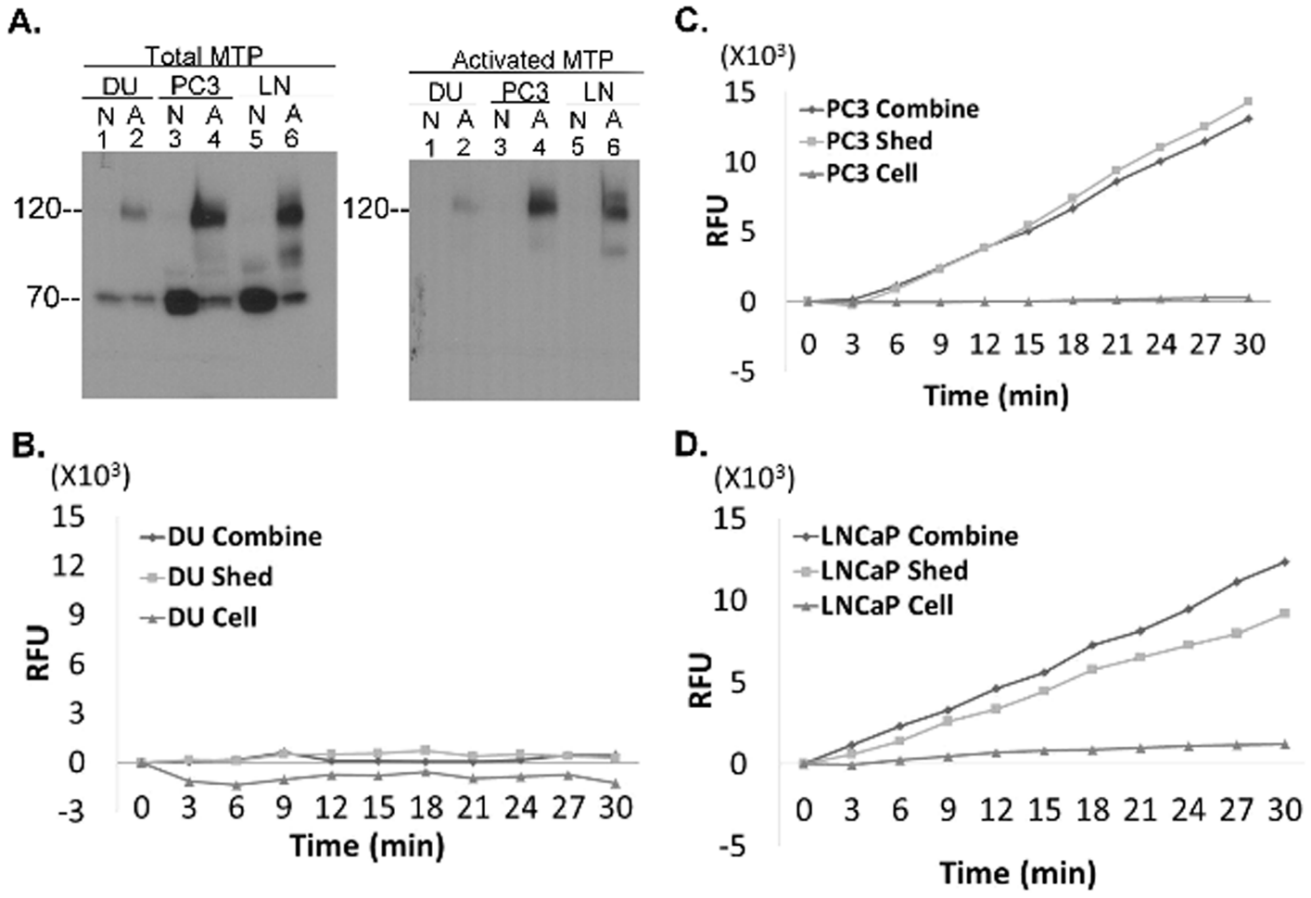
Human prostate cancer cell do not retain free, enzymatically active matriptase. *A*. The human prostate cancer cells, DU145 (DU), PC3 (PC3), and LNCaP (LN) were exposed to pH 6.0 (or control) buffer to induce matriptase activation. Cell lysates from pH 6-exposed cells (A, lanes 2, 4, and 6) and the non-activation control cells (N, lanes 1, 3, and 5) were analyzed for total matriptase species (left) using the mAb M24 and activated matriptase using the mAb M69 (right). *B*, *C*, and *D*. The human prostate cancer cells, DU145 (*B*), PC3 (*C*), and LNCaP (*D*) were treated with pH 6.0 buffer to induce matriptase activation. After neutralization, the combination of the cell and the shed fractions (combine), the cells fractions alone (cell), and the shed fraction alone (shed) were analyzed for tryptic activity. RFU stands for relative fluorescent units.

Matriptase is also expressed in prostate cancer, and the three most commonly used prostate cancer lines, DU145, PC3, and LNCaP all express matriptase although DU145 expresses much less than the other two lines (Fig. 4A, left, lanes 1, 3, and 5). All three lines activate matriptase in response to extracellular acid exposure as indicated by the presence of the 120 kDa matriptase-HAI-1 complex (Fig. 4A, left, lanes 2, 4, and 6), which is also detected when the samples are immunoblotted using the activated matriptase-specific mAb M69 (Fig. 4A, right, lanes 2, 4, and 6). Acid exposure of the cells also induced the shedding of tryptic activity into the extracellular milieu by PC3 and LNCaP cells but not by DU145 cells (Fig. 4 B–D). As with the breast cancer cell lines, the majority of the shed tryptic activity shed from, the PC3 and LNCaP cells, was confirmed to be associated with active matriptase by immunodepletion as described above (data not shown). The lack of detectable tryptic activity shed by the DU145 cells appears due to the low level of matriptase expression in this cell line. These data suggest that prostate cancer cells resemble breast cancer cells regarding the rapid HAI-1-mediated inhibition of cellular active matriptase and for the shedding of some levels of free active matriptase into the extracellular milieu.

**Figure 5:**
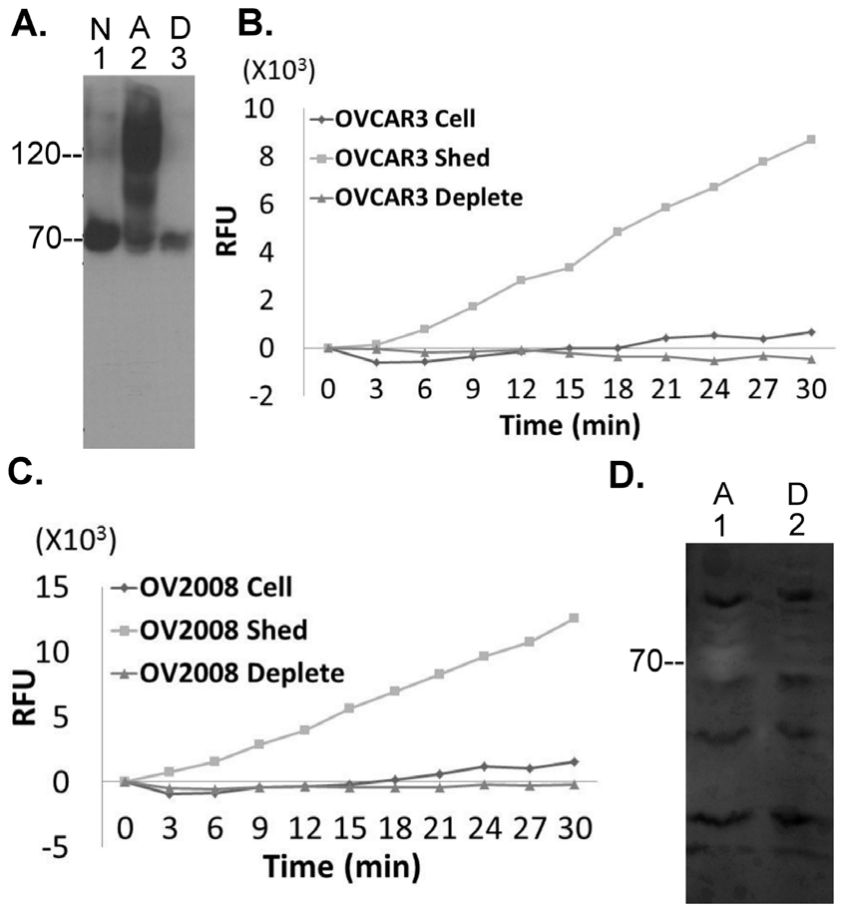
Human ovarian cancer cells do not retain enzymatically active matriptase. *A*. Human ovarian cancer cells OVCAR3 were induced to activate matriptase by pH 6.0 (or control) buffer. Cell lysates were immunodepleted with the mAb M69. Cell lysates of the non-activation control cells (N, lane 1), the pH 6 activated cells (A, lane 2), and the immunodepleted fraction (D, lane 3) were analyzed for matriptase species by Western blot using the M24 mAb. *B*. and C. The cells fractions (cell), the shed fractions (shed), and the activated matriptase-depleted shed fractions (deplete) of OVCAR3 (*B*.) cells and OV2008 cells (*C*.) were analyzed for tryptic activity. RFU stands for relative fluorescent units. *D.* OV2008 human ovarian cancer cells were induced to activate matriptase by pH 6.0 buffer. The shed fraction (lane 1) and the activated matriptase-depleted shed fraction (lane 2) were analyzed for matriptase gelatinolytic activity by gelatin zymography.

Two ovarian cancer cell lines that express matriptase, OCAR3 and OV2008, were also studied and revealed identical characteristics with respect to matriptase activation induced by exposure to pH 6.0 buffer with the rapid formation of activated matriptase-HAI-1 complexes, which were depleted by the mAb M69 (Fig. 5A). Neither cell line retained cell-associated tryptic activity but shed the activity into the buffer, which was verified to be derived from active matriptase by immunodepletion using the mAb M69 (Fig. 5 B and C). The shed matriptase exhibited gelatinolytic activity, which again was depleted by the mAb M69 (Fig. 5D).

### The majority of the activated matriptase shed by hematological cancer cells is shed as free active matriptase

**Figure 6:**
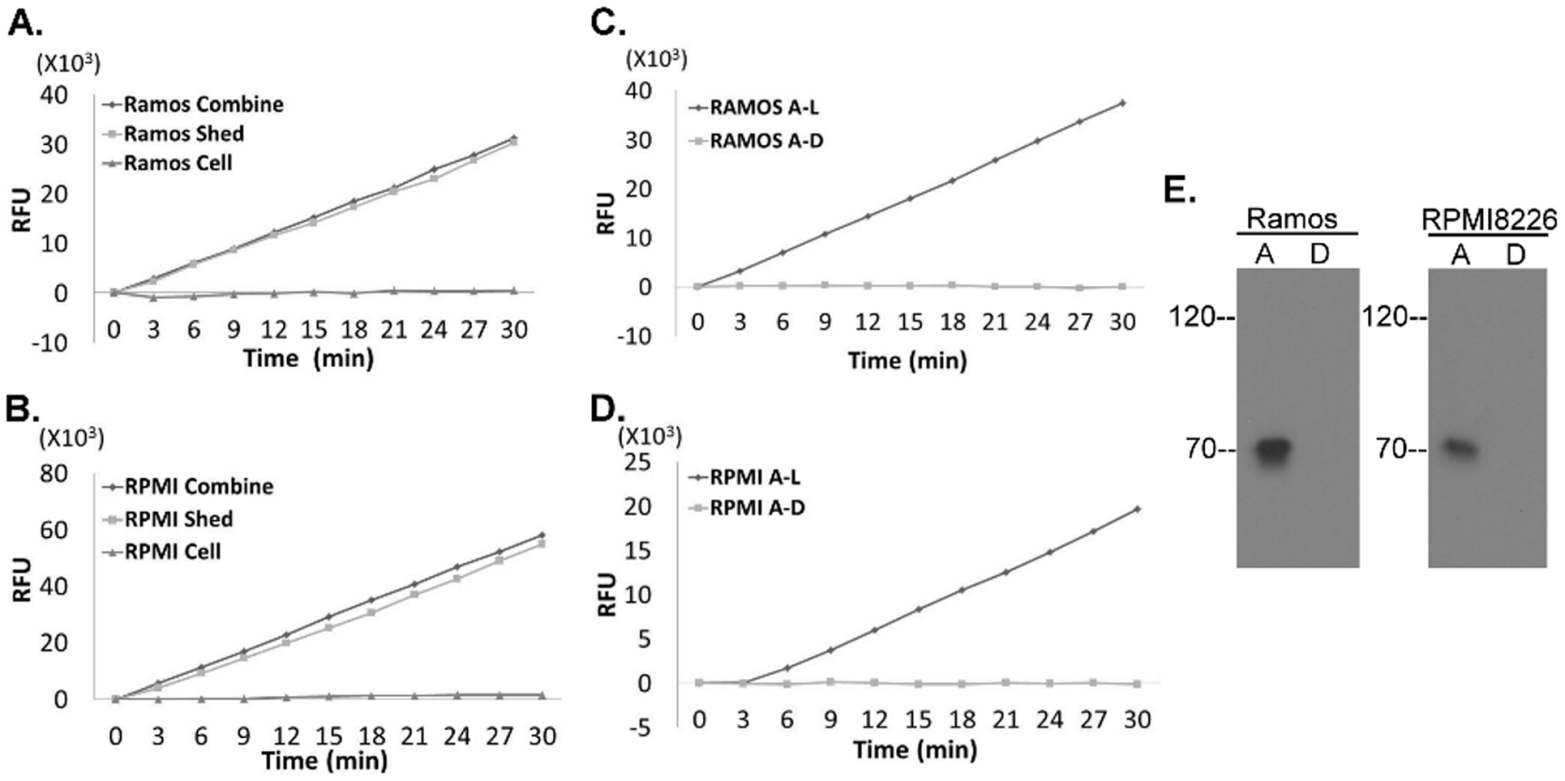
Human multiple myeloma cells and Burkitt lymphoma cells do not retain enzymatically active matriptase. *A* and *B.* Ramos human Burkitt lymphoma cells (*A*) and RPMI 8226 human multiple myeloma cells (*B*) were treated with pH 6.0 buffer to induce matriptase activation. After neutralization, the combination of the cell and the shed fraction (combine), the cell fraction alone (cell), and the shed fraction alone (shed) were analyzed for tryptic activity. *C* and *D*. Ramos (*C*) and RPMI 8226 cells (*D*) were treated with pH 6.0 buffer to induce matriptase activation. The shed fractions were immunodepleted using the activated matriptase mAb M69. The shed fractions (A–L) and the activated matriptase-depleted fractions (A–D) were analyzed for tryptic activity. (*E*) Detection of active matriptase with the activated matriptase mAb M69. Ramos human Burkitt lymphoma cells and RPMI 8226 multiple myeloma cells were treated with pH 6.0 buffer to induce matriptase activation. The shed fractions of Ramos cells and RPMI 8226 cells were immunodepleted using the activated matriptase mAb M69. The shed fractions (lanes A) and the activated matriptase-depleted fractions (lanes D) were analyzed by Western blot for activated matriptase using the mAb M69.

Matriptase is also expressed in hematological cancer cells but, in stark contrast to cells of epithelial origin, they express no or very low levels of HAI-1 [36], [37]. To explore the matriptase activation and shedding dynamics in cancers of this type we transiently exposed RPMI 8226 human multiple myeloma cells and Ramos Burkitt lymphoma cells to pH 6.0 buffer followed by adjustment to pH 8 with Tris buffer as we had done with the epithelial cancers. High levels of tryptic activity were detected in the mixture of the cells and the buffer (shed fraction) (Fig. 6A), and when the cells and shed fractions were separated by centrifugation, the tryptic activity was only detected in the shed fraction, and was not associated with the cells. Furthermore, the tryptic activity was completely removed when the shed fractions were depleted using the activated matriptase mAb beads, confirming that active matriptase is the source of the tryptic activity detected.

The non-adherent nature of both cancer cell lines made it possible for us to stimulate matriptase activation at very high cell density with 5×10^5^ cells per 0.2 ml of the pH 6.0 buffer. This ability in conjunction with the lack of HAI-1, allowed us to prepare shed fractions from the two hematological cancer cell lines with very high tryptic activity cleaving the substrates at a rate higher than 1,000 RFU/min. The high concentration of active matriptase present in these samples allowed us to detect this enzymatically active matriptase by immunoblotting using the activated matriptase mAb which revealed a band at expected size of 70-kDa (Fig. 6E, lanes A), the identity of which was confirmed by immunodepletion using the mAb beads (Fig. 6E, lanes D).

### Matriptase zymogen activation and shedding

**Figure 7:**
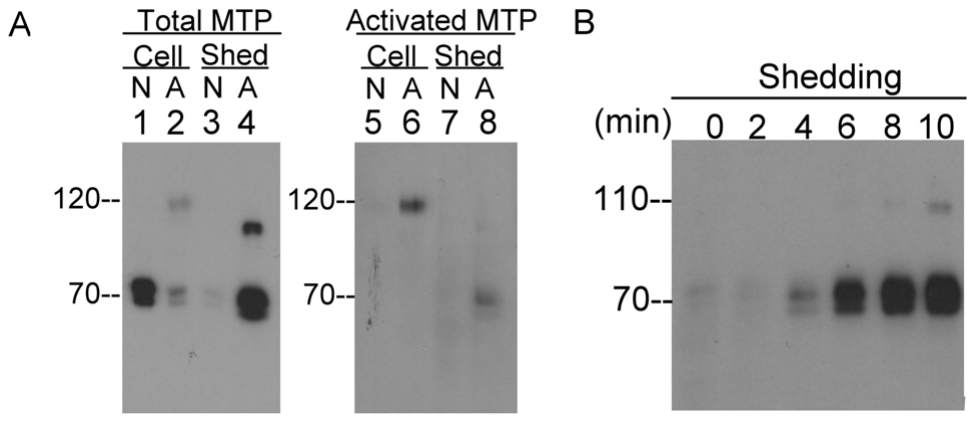
Matriptase shedding is tightly coupled with zymogen activation. *A.* HaCaT human keratinocytes were treated with pH 6.0 buffer (A) or PBS as a non-activation control (N) for 20 min. The cell lysates (Cell) and the shed fractions (Shed) were analyzed for total matriptase (Total MTP) using the mAb M24 and activated matriptase (Activated MTP) using mAb M69. *B.* HaCaT human keratinocytes were treated with pH 6.0 buffer to induce matriptase activation and shed fractions were collected at the indicated times thereafter and analyzed for matriptase using the mAb M24.

Matriptase is synthesized as a full-length 94-kDa species that undergoes N-terminal processing by cleavage within the SEA domain [38], [39]. As a result, mature matriptase is composed of two fragments, including a 16-kDa fragment that spans amino residue 1 through 149 and a 70-kDa fragment comprised of amino acid 150–855 covering the bulk of extracellular domains. The structure of the SEA domain holds both fragments together in a non-covalent manner on the cell membrane. The shedding of matriptase apparently requires new peptide bond cleavages, since our early study demonstrated that the N-terminal sequences for matriptase isolated from human milk were 190-SFVVTSVVAFPTDSKTVQRT-209 and 205-TVQRTQDNSCSFGLHARGVE-224 [33]. These sequences suggest that matriptase shedding requires the cleavage of a peptide bond between 189K-S190 or 204K-T205. The enhanced shedding of both the active and zymogen forms of matriptase following the induction of matriptase zymogen activation (Fig. 2) indicates that matriptase shedding may be tightly coupled with matriptase zymogen activation. In order to explore the functional link between matriptase zymogen activation and shedding, HaCaT human keratinocytes were exposed to pH 6.0 or PBS as a non-activation control for 20 min and the cell lysates and the conditioned buffer were analyzed for matriptase species using the total matriptase mAb M24 and the activated matriptase mAb M69 (Fig. 7A). For the non-activation control, matriptase remained in its 70-kDa zymogen form in the cell lysate (Fig. 7A lane 1), very little matriptase was shed 20 min (Fig. 7A lane 3), and no activated matriptase was detected (Fig. 7A lanes 5 and 7). For those cells exposed to pH 6.0 buffer, the levels of matriptase zymogen in the cell lysate was much lower than in the non-activation control and the 120-kDa matriptase-HAI-1 complex was readily detectable (Fig. 7A lane 2). A considerable amount of matriptase was shed into the buffer (Fig. 7A lane 4), consistent with the loss of cellular matriptase (Fig. 7A comparing lane 2 with lane 1). The apparent size of the shed “70-kDa” matriptase is slightly smaller than the cellular matriptase (Fig. 7A comparing lane 4 with lane 1), consistent with the involvement of proteolytic cleavage for shedding to occur. Active matriptase was also detected by the M69 activated matriptase-specific mAb in the shed fraction at around 70-kDa (Fig. 7A lane 8), consistent with the presence of free, active matriptase in the shed fraction. A 110-kDa matriptase species in the shed fraction was detected by the M24 total matriptase mAb (Fig. 7A, lane 4) but not by the M69 mAb, which we know from previous work is a matriptase-antithrombin (bovine) complex which results from active matriptase binding to antithrombin remaining on the cells from FBS in the culture medium [32]. The binding of antithrombin destroys the epitope recognized by the mAb M69 [12]. The kinetics of matriptase shedding was analyzed and revealed that matriptase shedding occurred as early as 4 min after acid exposure (Fig 7B), which is close to the timing of the onset of matriptase zymogen activation [27], [32]. These analyses reveal the functional linkage between matriptase shedding and zymogen activation, and support the rapid shedding of active matriptase to the extracellular milieu.

## Discussion

The question of whether cancer cells retain significant levels of the free, uncomplexed active form of matriptase or whether it is rapidly shed into the extracellular environment is important since the answer has major implications for its potential utility as a drug target and tumor bio-marker. Matriptase is expressed by essentially all epithelial cells within the body with the result that any strategy to capitalize on the dysregulation of matriptase that appears to be associated with poor outcome in multiple epithelial cancers [40], must distinguish in some way between the active and zymogen forms of the enzyme. If the active form of the enzyme is either shed from cells as soon as it is activated, or rapidly inhibited by HAI-1 as appears to occur in epithelial tissues, this would seem to limit, or at least complicate its utility as a target for catalytic inhibitors. Similarly, if all of the free active matriptase that is produced by some hematological cancers that do not express HAI-1 is immediately shed from the cells after activation, this would appear to limit its utility as marker for tumor imaging. This has led investigators to conduct studies (discussed below) that have address these questions and which have come to a variety of conclusions. In this study, we have tried to examine this issue in the light of this previous work and the data presented above lead us to conclude that very little if any free, active matriptase is retained on cancer cells after zymogen activation.

The matriptase transmembrane domain suggests that this serine protease could be synthesized in the rough endoplasmic reticulum, trafficking through the secretory pathway, orientated with the serine protease domain toward extracellular face of the plasma membrane. Matriptase detection on the cell surface and at cell-cell junctions with matriptase mAbs, the epitopes of which reside on the extracellular domains is consistent with this arrangement of the transmembrane domain of matriptase [35], [41]. The surface localization of matriptase does not, however, guarantee the presence of enzymatically active matriptase on cell surfaces. Matriptase can be shed in many forms, including the zymogen, enzymatically active forms, and activated forms in complex with HAI-1 or serpins [12], [42], [43]. Our current study and previous work suggest that only enzymatically inactive matriptase species, including the matriptase zymogen and activated matriptase in complexes with protease inhibitors are associated with cells. As mentioned above, there have, however, been several papers reporting the detection of free active matriptase on cell surfaces, some of which describe matriptase activity-targeting probes for *in vitro* and *in vivo* cancer cell detection. The discrepancy between our conclusion and these other reports may be attributed to the methods used for the detection of active matriptase and whether the approaches used in fact support the conclusions reached.

In a study by Deng et al., two matriptase-targeting molecular beacons containing a peptide linker with amino acid sequence of GRQSRAGC were prepared. The peptide linker is conjugated to a fluorescent dye on one end and attached to gold nanoparticle as a fluorescent quencher on the other end so that enzymatic cleavage of the linker results in a fluorescent signal [44]. Cleavage of this peptide linker is, however, not specific to matriptase, because many other proteases possess such activity. Neither the correlation between matriptase mRNA levels and the cleavage of the beacons by different cells, nor the inhibition of the cleavage of the beacons by aprotinin (a broad specificity inhibitor) is sufficient evidence to support the conclusion that the presence of enzymatically active matriptase on the cell surface is the sole protease responsible for cleavage. Cell-associated active matriptase was also reportedly detected by the use of a biotinylated peptide substrate-based chrolomethyl ketone (CMK) [45]. The substrate sequence of the CMK inhibitor was -Arg-Gln-Arg-Arg-, a sequence commonly used for the inhibitors and substrates of furin and related pro-protein convertases, but not for matriptase. Furthermore, the biotinylated CMK inhibitor can bind to matriptase zymogen, data provided in the same study, which clearly indicates the poor selectivity of the reagent between active proteases and their zymogens.

The presence of cell surface active matriptase was also reported in several matriptase-expressing human epithelial and carcinoma cells based on the detection of tryptic activity, followed by its inhibition using a matriptase single-chain antibody E2 for verification [24] and the direct detection of active matriptase on cell surfaces using the single-chain matriptase antibody A11 [25]. These single-chain matriptase antibodies were reported to potently inhibit active human matriptase with K*i*s of 10 pM and 35 pM, respectively. In order to reconcile the discrepancy between this and our current study we carefully compared the reagents and methods used. Although different synthetic fluorescent substrates (Gln-Ala-Arg *versus* Phe-Gly-Arg) were used, this substrate sequence difference should not explain the discrepancy since the P1 residue is the same, and the P2 residue is very similar. Unfortunately the substrate used by Darragh et al. [24] has been discontinued, and so we are unable to use it in our study for a direct comparison. If the substrates are comparable, the levels of the tryptic activity detected are very different between our study and Darragh's. Cleavage rates of the fluorescent substrate were between 5-15 relative fluorescent unites (RFU)/min in Darragh's study (these numbers were recalculated from RFU/second). In contrast, the cleavage rates in the shed fractions in the current study were around 200 RFU/min for breast cancer cells, around 100 RFU/min for prostate cancer cells, and around 150 RFU/min for ovarian cancer cells. For hematological cancer cells, the cleavage rates more than 1,000 RFU/min. Cleavage rates for cell fractions alone in the current study range from 0–20 RFU/min, similar to those in the Darragh's study. We do not consider the small increase in the fluorescence observed to be a positive signal since the kinetics for substrate cleavage by the cells alone fraction was not linear with significant fluctuations over time (data not shown). The tryptic activity in the Darragh's study was presented as a bar chart rather than as the kinetics of cleavage, with the result that we are unable to compare both studies on this aspect.

Although matriptase antibodies were used in both studies to demonstrate that the detected tryptic activity is derived from enzymatically active matriptase, there are differences in the characteristics of the matriptase antibodies and in the methods used for verification. We used an activated matriptase-specific monoclonal antibody, named M69, immobilized on Sepharose beads to immunodeplete the activated matriptase either as the free, uncomplexed form or in complex with HAI-1. The depletion of activated matriptase protein was verified by Western blot analyses using another matriptase mAb M24, which detects both the matriptase zymogen and activated matriptase. Furthermore, the recovered potent tryptic activity of the active matriptase dissociated from matriptase-HAI-1 complexes suggests that the matriptase tryptic activity is completely locked in the complexes with HAI-1. In Darragh's study, the single-chain matriptase antibodies used are potent catalytic inhibitors of matriptase as noted above. The concentration of the E2 antibody used to inhibit the cellular tryptic activity, however, was 200 nM, or about 20,000 times the K*i* of the E 2 matriptase antibody. In Nimishakavi study [46], 4 μM of a single-chain matriptase antibody was used to inhibit cell surface tryptic activity in human airway epithelial cells. Which antibody was used is not mentioned but this concentration would be 400,000 times the K*i* of the E2 antibody or more than 100,000 times the K*i* of the A11 antibody. The rationale underling the use of single-chain matriptase antibody at a concentration of 20,000 to 400,000 times the K*i* values to suppress cell surface tryptic activity was not described in either study. At these concentrations, the specificity of the matriptase inhibitory antibodies need to be verified if they are to be relied upon to implicate matriptase as the source of the tryptic activity. In spite of the discrepancy regarding the presence of active matriptase on cell surface of most cancer cells, the single-chain matriptase antibody A11 failed to detect active matriptase on SK-BR-3 breast cancer cells. This result is consistent with our conclusion that there is no free, active matriptase associated with the breast cancer cells in the current study (Fig. 3). SK-BR-3 breast cancer cells express significant levels of matriptase at the protein level as shown in our current and previous studies [5] as well as at the mRNA level in our previous study [5] and the study by others [47]. In Lebeau study [25], the lack of active matriptase on SK-BR-3 breast cancer cells was consistent with their hypothesis that low expression of matriptase and the ratio of matriptase relative to HAI-1 favors matriptase inhibition, however, the low matriptase expression in their SK-BR-3 cells suggests that either they are different from the ones we are using and those used by Welman et al [47]. We confirmed the identity of some of the cell lines used in this study by fingerprinting assay, and find that SK-BR-3 cells from the ATCC also express similar levels of matriptase. Alternatively, the lack of active matriptase detected in SK-BR-3 may indicate some sort of technical issues with the assay used in the Lebeau study. The immunofluorescence images of the active matriptase in these matriptase-expressing cancer cells seem to show that the signal is very strong, suggesting that they retain significant levels of active matriptase, which appears to be inconsistent with the weak tryptic activity detected in the Darragh study.

The shedding of matriptase appears to be mechanism that is tightly coupled with matriptase zymogen activation (Fig. 7). This could allow a proportion of the active matriptase to escape from HAI-1 inhibition through shedding in sufficiently rapidly manner that HAI-1 can not inhibit all of the active matriptase before it is shed. Shedding of the matriptase-HAI-1 complex requires an extra cleavage on HAI-1, a process that one would expect to be slower than matriptase shedding alone. Previously we demonstrated that human mammary epithelial cells begin to shed matriptase-HAI-1 complex around one hour after acid-induced activation, when the acid-exposed cells were returned to the culture medium and that the shedding proceeded for a couple of hours. In contrast to the acute matriptase zymogen activation induced by acid induction, matriptase zymogen activation can also be induced through other means with a delayed onset and at a slower rate. For example, LNCaP prostate cancer cells can be induced to activate matriptase in response to androgen treatment through an androgen receptor-dependent mechanism that involves de novo protein synthesis [48]. The onset of matriptase activation was 6 hr after androgen treatment. The resultant matriptase activation and associated shedding results in significant loss of cellular matriptase and the accumulation of shed matriptase zymogen and activated matriptase in the conditioned medium. In the context of the delayed and slow induction of matriptase activation, the extent of matriptase zymogen activation should be assessed by measuring the levels of activated matriptase both in the conditioned medium and in the cell lysate. Depending solely on determinations of the level of activated matriptase in the cell lysates to assess the degree of matriptase activation ongoing can be very misleading [49].

In summary, we have examined 10 human cancer lines from four different organs of origin for the fate of active matriptase following the induction of zymogen activation by extracellular acid exposure. These cancer cells all respond by activating matriptase to different extends. The active matriptase is rapidly inactivated by HAI-1 and remains on the cell surface of and inside carcinoma cells. Free active matriptase can be detected in the extracellular milieu of these cancer cells. Our study concludes that cancer cell associated free active matriptase and/or its proteolytic activity may not be the best target for the development of imaging or therapeutic strategies designed to capitalize on the dysregulation of the matriptase system in cancer.
